# Radiographers’ self-assessed level and use of competencies—a national survey

**DOI:** 10.1007/s13244-012-0194-8

**Published:** 2012-10-19

**Authors:** Bodil T. Andersson, Lennart Christensson, Ulf Jakobsson, Bengt Fridlund, Anders Broström

**Affiliations:** 1 Department of Nursing Science, School of Health Sciences, Jönköping University, Box 1026, SE-551 11 Jönköping, Sweden; 2 Center for Primary Health Care Research, Faculty of Medicine, Lund University, Lund, Sweden; 3 Department of Clinical Neurophysiology, University Hospital Linköping, Linköping, Sweden

**Keywords:** Competence, Assessment, Cross-sectional, Radiography, Nursing

## Abstract

**Objectives:**

To describe radiographers’ self-assessed level and use of competencies as well as how sociodemographic and situational factors are associated with these competencies, particularly related to work experience.

**Methods:**

A cross-sectional design was employed. Radiographers (n = 406) completed the self-administered 28-item questionnaire encompassing two dimensions: ‘Nurse-initiated care’ and ‘Technical and radiographic processes’. The level of competencies was rated on a 10-point scale and the frequency of use on a 6-point scale.

**Results:**

Most competencies received high ratings both in terms of level and frequency of use. In ‘Nurse-initiated care’ the competency ‘Adequately informing the patient’ was rated the highest, while ‘Identifying and encountering the patient in a state of shock’ and ‘Participating in quality improvement regarding patient safety and care’ received the lowest ratings. In ‘Technical and radiographic processes’ the highest rated competencies were ‘Adapting the examination to the patient’s prerequisites and needs’ and ‘Producing accurate and correct images’. The lowest frequency of use was ‘Preliminary assessment of images’.

**Conclusion:**

The main findings underline the radiographers’ high competency in both ‘Nurse-initiated care’ and ‘Technical and radiographic processes’. The lower rated competencies emphasise the importance of continuous professional education and quality improvement.

***Main Messages*:**

*• Assessing radiographers’ clinical competencies is fundamental for ensuring professional standards.*

*• Most competencies received high ratings both in the nursing and in the radiographic dimensions.*

*• The highest rated competencies focussed on information and adaptability to the patients needs.*

*• The lowest rated competencies focussed on encountering the patient in shock and image assessments.*

*• Age, years in present position and work place only explained a relatively small part of competency.*

## Introduction

Competency is an essential and challenging concept, continually discussed by health care professionals [1–4]. There is also a lack of consensus about the definition of competency, as well as how to measure it in clinical practice [5]. The definitions vary and, in particular, the simultaneous use of the terms ‘competency’ and ‘performance’ gives rise to confusion [6–8]. While [6] suggested a distinction between the two concepts, where competency indicates perceived skills and performance is actual behaviour and thus measurable. Competency has also been described as closely related to ‘being able to’ and ‘having the ability to do something’ [9]. Nevertheless, there is no agreement as to whether competency implies a greater level of ability or capacity compared to performance [9, 10]. Competency incorporates a combination of skills, knowledge, attitudes and the ability required in the performance of clinical practice [11]. A recent review emphasised a lack of clarity about the concept of competency and that it is more than skill, knowledge and attitude [12]. In addition, Benner [13, 14] defined general competency as the ability to perform a task with desirable outcomes. Correspondingly, Meretoja et al. [15] characterised competency as the performance of a task by the application of critical thinking, knowledge, technical and interpersonal skills.

Competency is closely related to patient safety and quality improvement, as well as to cost-effective health care [11, 16]. The increasing complexity and multiplicity of patient needs as well as changes in patient profiles have raised the requirements on competency in medical imaging departments, for example [17–19]. Hence, health care professionals must become aware of the need for optimal competencies in relation to patient care outcomes and the importance of developing a shared understanding of future competency requirements [20].

## Background

The recent advances in radiological technology and the changed radiographic process and nursing focus have influenced radiographers’ clinical competency [17, 21]. Both in- and out-patients require high quality care and support, to either detect a condition or provide relevant medical care (e.g., to a critically ill and unstable patient) [22]. It is also necessary to encounter both very young and elderly patients in a professional manner. In some specific clinical situations (e.g., mobile radiography services in primary health care or in homes for the elderly), no routine procedures are possible, which creates a need for flexibility [23]. Furthermore, the recent evolution of molecular imaging has increased the need for additional competency [24].

In most countries, registered nurses are responsible for nursing care, while a radiological technologist or corresponding professional is in charge of the radiological equipment [25]. However, in Sweden, highly educated specialist registered radiographers, who before 2001 were known as registered nurses in diagnostic radiology, have a unique position due to being responsible for the entire radiographic examination, nursing actions and medical technology, e.g., injections, catheterising and medical technical equipment [16]. There are no differences in training; the different names depend on the terminology of the academic degree. It is essential that radiographers continuously strive to improve their competency both in radiography and nursing care. In view of the recent work-related changes, self-assessments of competency are of importance since they may contribute to a basic understanding of the competency needs in clinical work today [26]. They can be useful for identifying organisational weaknesses, highlighting important educational needs and providing evidence for the development of preceptor courses [20, 27]. Lack of competency due to inadequate education and knowledge may increase the risk of injuries and put patient safety at risk [28]. Furthermore, competence assessments may promote individual professional development [29]. Assessing radiographers’ clinical competence is therefore of major importance in all medical imaging departments and a fundamental prerequisite for guaranteeing professional standards in both nursing care and radiography [30].

Despite the fact that self-assessment has been reported to be the most common form of competence evaluation [31], no studies defining the modern registered radiographers’ self-assessment of clinical competencies were identified, except the one Smith and Fisher conducted in 2011 [32], which focussed on registered nurses who had completed a short course in basic radiography. Accordingly, the aim of the present study was to describe radiographers’ self-assessed level and use of competencies, as well as how sociodemographic and situational factors are associated with these competencies, particularly in relation to work experience.

## Methods

### Design and setting

A national survey with a cross-sectional design set in 120 medical imaging departments at university (30 %), county (34 %) and district hospitals (36 %).

### Sample

The sample was drawn from a nationwide register administered by the Swedish Association of Health Professionals (SAHP), a trade union and professional organisation for radiographers, nurses, midwives and biomedical scientists. Based on the register, a computer randomly generated a list of 1,000 registered radiographers who were invited to participate. The inclusion criterion was that the participants should be clinically active and presently working as radiographers. At the time of the study, 2,167 of the registered radiographers and diagnostic radiology nurses listed in the register were members of SAHP, of whom 1*,*772 (82 %) met the inclusion criterion.

### Questionnaire

The Radiographers’ Competence Scale (RCS), a self-administered 28-item questionnaire, was used to collect data [33]. The questionnaire encompassed two dimensions of radiographers’ competencies: ‘Nurse-initiated care’ (18 items) and ‘Technical and radiographic processes’ (10 items). Each item represented a competence and was answered by means of a two-part scale, one of which focussed on the value placed on the radiographers’ competence and the other on the frequency of its use. Radiographers responded to statements by rating the level of competence on a 10-point scale (1–10)*,* where 1 was the lowest and 10 the highest grade. The frequency of using the competence was rated by means of the following response alternatives: “never used”, “very seldom used”, “sometimes used”, “often used”, “very often used” and “always used”. The questionnaire has been tested for validity and reliability, demonstrating acceptable psychometric properties (Cronbach’s alpha, 0.87) [33].

### Data collection

A link to the questionnaire was e-mailed to 1*,*000 participants in late autumn 2010. Background questions concerning age, sex, present position, basic education, highest academic level and work place were included in the questionnaire. An accompanying letter was distributed, containing information about the study and ethical aspects. A first reminder was sent after 1 week and a second after 2 weeks.

### Data analysis

Four groups were created based on ‘years in present position’ (0–5 years; 5–15 years; 15–25 years; >25 years). In the results, the participants included in the different groups are presented as radiographers with short-, medium- and long-term as well as the longest period of work experience. The groups were then compared regarding the sample characteristics, namely level and use of competence. Analyses were performed using the chi-square test when comparing competencies between groups. For nominal data, the one-way ANOVA test was used for comparison of the four groups, i.e., six comparisons, with regard to the level and use of competencies. Spearman’s rank order correlation was employed in order to perform correlations for the sum of the variables in the two dimensions of the RCS. Consequently, the mean score for each dimension was constituted by the 18 and 10 competencies in each of the two dimensions of the RCS (i.e., ‘Nurse-initiated care’ and ‘Technical and radiographic processes’). The mean scores were employed to compare the relationship between the level of competencies and the frequency of using them in the different groups. A p-value below 0.05 was considered significant except in the post-hoc analyses. Due to multiple comparisons, a reduced p-value of <0.008, according to the Bonferroni method [34], was applied to control for the risk of mass significance. Two linear regression analyses were performed to analyse the relationship between the dimension scores and the characteristics of the sample (i.e., age, years in present position and work place). All data were computerised and analysed using SPSS 18.0 for Windows (SPSS Inc., Chicago, IL, USA).

### Ethical considerations

This study was conducted in accordance with the principles outlined in the Declaration of Helsinki [35]. It was not considered necessary to apply for permission from the regional research ethics committee, but the study adhered to the ethical guidelines for nursing research [36]. Participants were provided with written information about the study and informed that participation was voluntary, that the data would be treated confidentially and that they could withdraw at any time. It is impossible to associate any answer with a specific participant. Completing the questionnaire implied informed consent.

## Results

### Sample characteristics

A total of 406 clinically active radiographers, 88 % of whom were women, completed the questionnaire, a response rate of 41 %. Their mean age was 47 years (SD 10.6, range 22–66). Of the total sample, 73 % had a basic education as a radiographer and 27 % as a registered nurse in diagnostic radiology. The academic level was higher among those with fewer years of experience. Besides a bachelor degree, 7 % had academic studies at the master or postgraduate level. Almost 90 % had a position as a registered radiographer and 9 % a management position. The majority (54 %) had short- and medium-term work experience. Sex, present position and highest academic level did not differ between the groups. Characteristics of the sample are presented in Table 1.

**Table 1:** Characteristics of the sample (n = 406)

	Years in present position					
	Total	0–5 years (I)	>5–15 years (II)	>15–25 (III)	>25 (IV)	
n (%)^a^	378 (93.0)	n = 111 (27.3)	n = 110 (27.1)	n = 61 (15.0)	n = 96 (23.6)	p-value
Age, m (SD)	47 (10.6)	39 (11.1)	46 (8.8)	50 (6.8)	56 (4.8)	<0.001^*^
Sex, n (%)^b^						0.270^*^
Female	331 (87.6)	97 (87.7)	92 (83.6)	53 (86.9)	89 (92.7)	
Male	47 (12.4)	14 (12.6)	18 (16.4)	8 (16.1)	7 (7.3)	
Present position, n (%)^c^						0.036^**^
Reg. radiographer	306 (81.6)	89 (81.7)	81 (74.3)	52 (85.2)	84 (87.5)	
Reg. radiographer with specialisation	29 (7.7)	7 (6.4)	15 (13.8)	4 (6.6)	3 (3.1)	
Reg. nurse	7 (1.9)	0 (0.0)	5 (4.6)	1 (1.6)	1 (1.0)	
Reg. radiographer/nurse in management position	33 (8.8)	13 (11.9)	8 (7.3)	4 (6.6)	8 (8.3)	
Basic education, n (%)^b^						<0.001^**^; A, B, E, F
Reg. radiographer	276 (73.0)	96 (86.5)	55 (50.0)	39 (63.9)	86 (89.6)	
Reg. nurse in diagnostic radiology	100 (26.5)	15 (13.5)	53 (48.2)	22 (36.1)	10 (10.4)	
Other	2 (0.5)	0 (0.0)	2 (1.8)	0 (0.0)	0 (0.0)	
Highest academic level, n (%)^d^						0.394^**^
High school diploma	86 (33.9)	11 (11.8)	16 (20.8)	19 (63.3)	40 (74.1)	
Bachelor degree	151 (59.4)	77 (82.8)	52 (67.5)	9 (30.0)	13 (24.1)	
Master degree	14 (5.5)	3 (3.2)	8 (10.4)	2 (6.7)	1 (1.9)	
Postgraduate level	3 (1.2)	2 (2.2)	1 (1.3)	0 (0.0)	0 (0.0)	
Working place, n (%) ^e^						<0.001^**^; B, C, E
University hospital	111 (29.8)	41 (36.9)	30 (28.0)	10 (16.7)	30 (31.6)	
County hospital	126 (33.8)	40 (36.0)	45 (42.1)	21 (35.0)	20 (21.1)	
District hospital	136 (36.5)	30 (27.0)	32 (29.9)	29 (48.3)	45 (47.4)	

^a^Missing = 28 (7.0)

^b^Missing = 28 (6.9)

^c^Missing = 31 (7.6)

^d^Missing = 152 (37.4)

^e^Missing = 33 (8.1)

Significant differences between: A. I–II; B. I–III; C. I–IV; D. II–III; E.II–IV; F. III–IV

^*^One-way ANOVA test

^**^Chi square test

p-value 0.008 was used for post-hoc analysis

Sign

### Self-assessed level of professional competencies

Table 2 illustrates that all four groups rated their professional competencies as high. The levels increased in line with years in present position. In ‘Nurse-initiated care’, the highest rated competency was ‘Adequately informing the patient’, while the lowest were ‘Identifying and encountering the patient in a state of shock’ and ‘Participating in quality improvement regarding patient safety and care’. In ‘Technical and radiographic processes’ the highest rated competency was ‘Adapting the examination to the patient’s prerequisites and needs’, whereas the lowest was ‘Preliminary assessment of images’.

**Table 2:** The level of radiographers’ competencies (n = 406). Comparison between groups with different number of years in present position

	Years in present position				
	0–5 years (I)	>5–15 years (II)	>15–25 years (III)	>25 years (IV)	
Nurse-initiated care	M (SD)	M (SD)	M (SD)	M (SD)	*p-*value
Carrying out doctors’ instructions	8.57 (1.51)	8.91 (1.48)	8.97 (1.23)	9.21 (1.07)	0.011; C
Applying ethical guidelines	8.64 (1.33)	8.74 (1.16)	8.75 (1.24)	8.99 (1.01)	0.199
Adequately informing the patient	9.01 (1.02)	9.10 (0.83)	9.11 (0.92)	9.43 (0.65)	0.005; C, E
Guiding and educating the patient	8.76 (1.15)	8.84 (1.21)	8.98 (1.31)	9.22 (0.95)	0.027; C
Empowering the patient by involving him/her in the examination and treatment	8.63 (1.36)	8.69 (1.13)	8.95 (1.17)	9.13 (0.98)	0.010; E
Guiding the patient’s relatives	8.20 (1.78)	8.29 (1.46)	8.42 (1.25)	8.76 (1.33)	0.050; C
Encouraging and supporting the patient	8.81 (1.21)	8.81 (1.08)	8.97 (1.06)	9.19 (0.87)	0.040
Protecting the patient’s integrity	9.00 (1.19)	9.19 (0.97)	9.22 (0.89)	9.33 (0.95)	0.147
Alleviating the patient’s anxiety	8.64 (1.20)	8.76 (1.13)	8.93 (1.08)	9.14 (1.04)	0.012; C
Judging the risk of leaving the patient unattended	8.38 (1.54)	8.82 (1.21)	8.97 (1.02)	9.08 (0.99)	0.001; C
Observing and monitoring the patient	8.26 (1.59)	8.35 (1.78)	8.23 (1.52)	8.65 (1.41)	0.281
Identifying and encountering the patient in a state of shock	6.84 (2.11)	7.01 (1.86)	7.02 (2.15)	7.58 (1.94)	0.067
Identifying pain and pain reactions	7.85 (1.51)	7.99 (1.42)	7.73 (1.66)	8.34 (1.44)	0.055
Collaborating with internal and external colleagues	8.57 (1.18)	8.70 (1.01)	8.72 (1.23)	8.76 (1.23)	0.687
Collaborating with other internal and external professionals	8.39 (1.42)	8.56 (1.25)	8.45 (1.48)	8.62 (1.30)	0.629
Supervising and training colleagues and other co-workers	7.90 (1.67)	8.04 (1.33)	8.14 (1.56)	8.62 (1.23)	0.004
Reporting to colleagues and other professionals, internal as well as external	8.32 (1.47)	8.33 (1.39)	8.42 (1.57)	8.67 (1.24)	0.270
Participating in quality improvement regarding patient safety and care	7.55 (1.83)	7.39 (1.74)	7.15 (1.96)	7.91 (1.50)	0.048; F
M (SD)	8.28 (1.10)	8.46 (0.83)	8.45 (1.01)	8.85 (0.80)	<0.001
Technical and radiographic processes					
Organising and planning***,*** taking account of the clinical situation	8.06 (1.36)	8.35 (1.15)	8.42 (1.08)	8.76 (0.99)	<0.001; C
Responsibility for preparing the medico-technical equipment	8.52 (1.22)	8.78 (1.02)	8.69 (1.19)	9.01 (1.13)	0.022; C
Independently planning and preparing work on the basis of existing documentation	8.21 (1.23)	8.55 (1.32)	8.61 (1.10)	9.02 (0.92)	<0.001; C, E
Prioritising patients in the work flow	8.09 (1.80)	8.17 (1.52)	8.58 (1.24)	8.78 (1.28)	0.004; C, E
Adapting the examination to the patient’s prerequisites and needs	8.81 (1.11)	8.91 (1.35)	9.07 (1.29)	9.25 (0.99)	0.052
Minimising radiation doses for patient and staff	8.61 (1.33)	8.71 (1.46)	8.88 (1.13)	9.03 (1.05)	0.104
Producing accurate and correct images	8.83 (1.12)	8.75 (1.35)	8.93 (1.08)	9.22 (0.89)	0.021; E
Evaluating the quality of the medical image in relation to the referral and the question stated therein	8.74 (1.01)	8.75 (1.35)	8.93 (1.00)	9.21 (0.91)	0.008; C, E
Optimising the quality of the image	8.26 (1.49)	8.36 (1.37)	8.52 (1.47)	8.89 (1.03)	0.007; C, E
Preliminary assessment of images	8.06 (1.38)	8.06 (1.57)	8.54 (1.15)	8.73 (1.46)	0.053
M (SD)	7.92 (0.93)	8.04 (0.90)	8.13 (0.87)	8.47 (0.75)	<0.001

Significant differences between: A. I–II; B. I–III; C. I–IV; D. II–III; E.II–IV; F. III–IV

One-way ANOVA test

Reduced p*-*value < 0.008 was used for post-hoc analyses

Differences related to number of years in present position are presented in Table 2. In ‘Nurse-initiated care’ those with a short period of work experience rated, for example, ‘Alleviating the patient’s anxiety’ and ‘Judging the risk of leaving the patient unattended’ lower than those with the longest period of work experience (p < 0.001). Those with short- and medium-term experience scored ‘Adequately informing the patient’ lower compared to those with the longest period (p < 0.001). In ‘Technical and radiographic processes’, for example ‘Organising and planning taking account of the clinical situation’ and ‘Independently planning and preparing work on the basis of existing documentation’, were rated higher by those with the longest period of work experience (p < 0.001). The ratings were similar in ‘Prioritising patients in the work flow’, ‘Producing accurate and correct images’ and ‘Optimising the quality of the image’.

### Self-assessed use of professional competencies

Table 3 illustrates how frequently the radiographers used their professional competencies in relation to the number of years of work experience. The highest rated competence in ‘Nurse-initiated care’ was ‘Adequately informing the patient’, while the lowest was ‘Identifying and encountering the patient in a state of shock’. ‘Participating in quality improvement regarding patient safety and care’ as well as ‘Reporting to colleagues and other professionals, internal as well as external’ had low ratings in all groups. All competencies in ‘Technical and radiographic processes’ were highly rated. Producing accurate and correct images’ and ‘Adapting the examination to the patient’s prerequisites’ were rated as the most frequently used.

**Table 3:** The use of radiographers’ competencies. Comparison between groups with different number of years in present position (%)

	Years in present position												
	0–5 years (I)			>5–15 years (II)			>15–25 years (III)			>25 years (IV)			*p-*value
Responses in the RCS*	3	4	5–6	3	4	5–6	3	4	5–6	3	4	5–6	
Nurse-initiated care													
1. Carrying out doctors’ instructions	20	62	-	9	74	-	8	71	-	9	79	-	0.233
2. Applying ethical guidelines	5	14	76	8	24	66	13	12	71	3	14	81	0.031; E
3. Adequately informing the patient	2	7	86	3	5	92	-	7	92	1	2	97	0.066
4. Guiding and educating the patient	7	12	75	6	10	80	7	3	84	4	10	85	0.436
5. Empowering the patient by involving him/her in the examination and treatment	6	14	74	5	17	76	7	16	71	2	10	88	0.164
6. Guiding the patient’s relatives	19	15	54	32	16	43	18	23	53	19	16	63	0.011; E
7. Encouraging and supporting the patient	14	25	55	12	21	66	12	16	71	4	14	81	0.004; C,E
8. Protecting the patient’s integrity	3	13	79	4	7	88	2	10	87	3	9	87	0.406
9. Alleviating the patient’s anxiety	12	23	60	6	15	77	7	13	77	4	15	80	0.016; C
10. Judging the risk of leaving the patient unattended	23	17	52	18	14	61	15	15	64	10	15	70	0.118
11. Observing and monitoring the patient	19	22	47	19	19	51	21	18	49	17	9	65	0.465
12. Identifying and encountering the patient in a state of shock	29	10	20	32	14	19	28	10	21	22	9	33	0.179
13. Identifying pain and pain reactions	23	30	36	23	28	40	28	26	38	16	30	48	0.115
14. Collaborating with internal and external colleagues	5	17	70	14	14	69	15	26	56	12	16	71	0.192
15. Collaborating with other internal and external professionals	10	19	65	13	16	62	23	30	44	17	14	65	0.039; B
16. Supervising and training colleagues and other co-workers	32	14	46	29	24	38	16	26	43	26	8	59	0.025; E
17. Reporting to colleagues and other professionals, internal as well as external	25	18	33	36	22	29	36	16	39	23	21	42	0.176
18. Participating in quality improvement regarding patient safety and care	24	20	31	42	17	25	39	16	25	33	21	35	0.088
Technical and radiographic processes													
19. Organising and planning taking account of the clinical situation	11	23	63	10	18	67	7	26	64	2	19	78	0.048; E
20. Responsibility for preparing the medico-technical equipment	3	18	76	5	13	80	3	18	77	1	12	84	0.363
21. Independently planning and preparing work on the basis of existing documentation	9	19	68	6	15	74	3	15	77	4	8	85	0.015; C
22. Prioritising patients in the work flow	8	23	60	9	24	60	12	18	62	5	16	77	0.005; E
23. Adapting the examination to the patient’s prerequisites and needs	5	14	78	5	14	76	5	10	79	2	7	88	0.142
24. Minimising radiation doses for patient and staff	4	10	79	8	15	69	5	10	77	-	12	81	0.048; E
25. Producing accurate and correct images	2	5	86	4	11	79	3	15	77	1	5	92	0.106
26. Evaluating the quality of the medical image in relation to the referral and the question stated therein	2	8	84	4	12	80	2	12	82	4	8	85	0.234
27. Optimising the quality of the image	5	14	73	13	11	70	13	20	62	5	6	84	0.014; E, F
28. Preliminary assessment of images	15	14	64	8	26	55	5	12	75	8	15	73	0.021; E

*Responses from the RCS; 3–6 are used in the table and 1–2 are veiled

1 = never used; 2 = very seldom used; 3 = sometimes used; 4 = often used; 5 = very often used; 6 = always used

Significant differences between: A. I–II; B. I–III; C. I–IV; D. II–III; E.II–IV; F. III–IV

One-way ANOVA test.

Reduced *p-*value < 0.008 was used for post-hoc analyses

Differences related to years in present position are presented in Table 3. In ‘Nurse-initiated care’, those with a short period of work experience used ‘Collaborating with other internal and external professionals’ more frequently compared to those with long-term experience (p < 0.001). The latter used ‘Applying ethical guidelines’, ‘Guiding the patient’s relatives’, ‘Encouraging and supporting the patient’ and ‘Alleviating the patient’s anxiety’ more frequently compared to those with a short period of work experience (p < 0.001). In ‘Technical and radiographic processes’, those with long-term experience used ‘Organising and planning taking account of the clinical situation’, ‘Prioritising patients in the work flow’, ‘Minimising radiation doses for patient and staff’, ‘Optimising the quality of the image’ and ‘Preliminary assessment of images’ more often than those with short experience (p < 0.001).

### Variables associated with professional competencies

The total score for ‘Nurse-initiated care’ correlated significantly with age (r = 0.265; p < 0.001) and years in present position (r = 0.217; p < 0.001). The total score for ‘Technical and radiographic processes’ also correlated significantly with age (r = 0.278; p < 0.001) and years in present position (r = 0.287; p < 0.001). Tables 4 and 5 present the regression models. The competence level was significantly associated with age in ‘Nurse-initiated care’ and ‘Technical and radiographic processes’ as well as with the total score of the RCS (Table 4). The use of competency was significantly associated with years in present position in ‘Technical and radiographic processes’ and the total score of the RCS, but not in ‘Nurse-initiated care’ (Table 5).

**Table 4:** Variables associated with ‘Nurse-initiated care’ and ‘Technical and radiographic processes’ related to the level of competence among radiographers (n = 406)

Dimension/scales of the dependent variable	Model/independent variable	B	95.0 % CI for B	p-value
Nurse-initiated care (adjusted R^2^ for model: 0.080)	Age	0.019	0.007 to 0.031	<0.003
	Years in present position	0.008	−0.002 to 0.018	0.111
	Work place	−0.187	−0.404 to 0.030	0.090
Technical and radiographic processes (adjusted R^2^ for model: 0.066)	Age	0.016	0.005 to 0.028	<0.005
	Years in present position	0.007	−0.002 to 0.017	0.138
	Work place	−0.074	−0.283 to 0.136	0.488
Radiographers’ competence scale (RCS) (adjusted R^2^ for model: 0.070)	Age	0.016	0.005 to 0.028	<0.007
	Years in present position	0.007	−0.003 to 0.017	0.154
	Work place	−0.187	−0.402 to 0.027	0.087

Variables in the regression analysis: age, years in present position and work place

**Table 5:** Variables associated with ‘Nurse-initiated care’ and ‘Technical and radiographic processes’ related to the use of competence among radiographers (n = 406)

Dimension/scales of the dependent variable	Model/independent variable	B	95.0 % CI for B	p-value
Nurse-initiated care (adjusted R^2^ for model: 0.025)	Age	0.004	−0.005 to 0.014	0.396
	Years in present position	0.007	0.000 to 0.015	0.063
	Work place	−0.118	−0.286 to 0.050	0.168
Technical and radiographic processes (adjusted R^2^ for model: 0.020)	Age	−0.002	−0.010 to 0.007	0.687
	Years in present position	0.009	0.002 to 0.016	−0.010
	Work place	0.066	−0.092 to 0.223	0.413
Radiographers’ competence scale (RCS) (adjusted R^2^ for model: 0.015)	Age	0.001	0.010 to −0.007	0.850
	Years in present position	0.007	0.002 to 0.016	0.045
	Work place	−0.069	−0.229 to 0.090	0.393

Variables in the regression analysis age, years in present position and work place

## Discussion

The main findings of this study were that radiographers assessed their overall competency and use of individual competencies as being on a high level. We found that both the level and use of several competencies differed in line with the number of years in present position. However, the regression models using age, years in present position and work place only explained a relatively small part of competency, which indicates a multidimensional situation including other factors of importance.

The radiographers considered that they had high competence regarding patient information. ‘Adequately informing the patient’ was an item with a high mean score, irrespective of work experience. The importance of this competence is confirmed by other studies [27, 37]. One must bear in mind that the patient is unknown to the radiographer and rarely encounters the same person again, which makes the encounter particularly transitory and the informative part significant [25, 38, 39]. Studies focussing on nursing in general have emphasised adequate information as an important competence [13, 29] and a requirement for increased patient participation [40]. However, others have found teaching-coaching to be a poorly rated competence among operating room nurses, who also encounter the patient for a short period of time, compared to other nurses [41]. Equally, nurses in a clinical position rate teaching-coaching activities low compared to their counterparts in management positions [42]. Information about radiographic procedures is highly important [43], especially from the patient perspective. It should be based on a dialogue, adjusted to the situation, and can therefore be provided in many different ways (i.e., oral, written and interactive media) [44, 45]. The information can contain both counselling and teaching with the aim of guiding the patient through the radiographic process and increasing her/his coping skills [46, 47]. All medical imaging departments have a high-tech environment with a great complexity of examinations and treatments, as well as a limited duration of the encounter between the radiographer and patient. Recent technical developments, and especially the evolution of molecular imaging [24], demand new studies focussing on both patients’ and radiographers’ views as well as on how to provide information.

The radiographers in the present study scored low on ‘Identifying and encountering the patient in a state of shock’, which may demonstrate that it is a complex clinical situation that requires relevant education and many years of experience. Radiographers must have the ability to detect changes in the patient’s condition at an early stage, to monitor and follow the course of events and decide when to terminate an examination [16]. Being vigilant in emergency situations can be of vital importance and involves competencies and requirements based on skill and flexibility. Our findings revealed that the level was lowest among those with short experience, which indicates that the length of work experience may play a crucial part in relation to this competence. Furthermore, the radiographers in the present study considered that they had low competence in ‘Identifying pain and pain reactions’. Many patients experience pain during their hospital stay, and departments often have inadequate pain assessment routines [48]. It is known that several radiographic examinations are associated with pain; thus the radiographer plays an important role in pain management by ensuring that pain is identified and reduced before and during the examination. To minimise pain, patients with a hip fracture, for example, are given higher priority at the medical imaging department [49]. From the patient perspective, even if an examination only takes a few minutes, one should not have to suffer severe pain. The radiographer is responsible for the entire procedure and can be seen as the patient’s advocate. Radiographers’ ability to relieve pain is therefore of high priority both during education and in clinical practice.

Management of situations is a vital competence [31, 40, 50]. We found that the radiographers considered themselves highly competent in ‘Adapting the examination to the patient’s prerequisites and needs’. A radiographer often has to perform examinations on patients who are unable to play an active part in the procedure (e.g., critically injured patients, those suffering from dementia or orthopaedic patients in plaster). These situations demand a high degree of flexibility and the ability to improvise. The competence to adapt can, however, be seen as contradictory in relation to the self-assessed low competencies pertaining to ‘Identifying and encountering the patient in a state of shock’ and ‘Identifying pain and pain reactions’. Radiographers often face anaphylactic reactions in relation to contrast medium and should therefore be confident in managing patients in a state of shock. However, the low score on both of these competencies might be understood as a lack of knowledge related to other medical causes of shock (e.g., severe internal bleeding leading to haemodynamic reactions) as well as the absence of assessment and pharmacological treatment of pain. On the other hand, a radiographer can encounter a critically injured patient without extensive in-depth information about the patient’s medical condition, which might further complicate the situation. However, proper professional training from the beginning of a radiographer’s education in relation to these topics is therefore highly important. Bearing this in mind, the education system, both on basic and advanced levels, as well as quality improvement projects in clinical practice should place more emphasis on these important topics.

An interesting finding was that the radiographers in the present study considered themselves to have low competence in ‘Participating in quality improvement regarding patient safety and care’. This is in line with other studies regarding competence [31, 40, 50] and can be understood in the light of the rapid development of high technology, increased national requirements in radiation safety and patient care, as well as economic demands [51].

We found that both age and years in present position correlated significantly with the competencies in ‘Nurse-initiated care’ and ‘Technical and radiographic processes’ as well as with the RCS as a whole. However, the R^2^ values in the linear regressions were very low, and years in present position and work place were not significant in the two dimensions or in relation to the RCS as a whole. When examining the literature, no previous studies regarding factors associated with radiographers’ self-assessed competence were found. According to Benner [13] and Dreyfus and Dreyfus [52], five levels of professional pathway ‘from novice to expert’ are described as the basis for gaining increased skill and competencies. The progress from a novice to an expert is almost always combined with many years of experience. However, the number of years of experience does not automatically mean that the individual will reach the competent, proficient or expert levels [13]. The lack of association between self-assessed competence and age, years in present position and work place in the present study indicates that there are several other variables that should be taken into consideration, such as the radiographer’s own level of knowledge and/or competence as well as the use of evidence-based knowledge at the actual department. An experienced radiographer working at a university hospital (i.e., using evidence-based knowledge) with in-depth knowledge of both ‘Nurse-initiated care’ and ‘Technical and radiographic processes’ might have better ability to evaluate lack of competence compared to a newly qualified radiographer with little experience working at a district hospital. A multi-rater feedback (i.e., 360° feed-back) could be used as a possible additional description of radiographers’ competencies [53].

Effects of the education system or the individuals’ habitual behaviour in clinical practice might be other possible factors of importance. Even if radiographers have theoretical knowledge, routines in the clinical situation are often based on unconscious habitual behaviours [54], which might influence the newly qualified radiographer’s opportunity to implement her/his theoretical knowledge in clinical practice, thus affecting both her/his self-assessed level and use of competence. However, a habitus can also be used consciously, toward a specific goal [55], to encourage improvement. From a methodological perspective, the RCS is a newly developed instrument showing good validity and reliability [33]. We decided to divide our relatively large national sample into four groups to describe the progress of competency. The variation regarding years in present position was good, despite the fact that one of the four groups had a smaller number of participants. Most participants had a university/high school education. However, no comparisons were made between participants with different levels and types of education or frequencies of use since so few of them had higher education (i.e., master or doctoral degree). There was a predominance of female participants, 88 %, in the present study. This reflects the sex distribution among Swedish radiographers and may therefore not be seen as a bias for the result. Structural equation modelling could be another statistical method for assessing associations between different variables and competencies. Besides, future research could focus on comparisons between self-assessment and outside assessments, based on health-care personnel and/or patients regarding clinical competence among radiographers.

### Relevance to clinical practice

Medical imaging departments are central in the health-care service. Self-assessments of competence, especially using a validated tool such as the RCS, are highly important for clinical practice. Results from competence assessments could be used in areas such as patient safety, planning and evaluating competence development, as well as in management. The two dimensions in the RCS, ‘Nurse-initiated care’ and ‘Technical and radiographic processes’, illuminate a relationship between technological and caring competence and provide a more detailed picture of radiographers’ clinical competencies. This can contribute to a baseline in terms of educational needs (i.e., in basic and further education, as well as in clinical practice), but also for evaluating quality improvements related to radiographers’ clinical work situation. Moreover, it could also be valuable for radiographers to reflect on their own competence, role and development possibilities since a competence assessment may be a rewarding process as it provides information about less obvious matters in clinical practice. The information obtained may also help safeguard the patients’ health and emphasise nursing issues and not merely the technological aspects of the procedures. Furthermore, knowledge derived from competence assessments may be used in different areas of professional development and education areas in various health care settings. Knowledge about radiographers’ clinical competence can contribute to the development of new routines and a more individualised approach to improving their work situation. Focussing on different competencies, e.g., the findings that the radiographers considered themselves least competent in ‘Preliminary assessment of images’, may highlight the fact that assessment of images is still not a common task for Swedish radiographers. An important implication for clinical practice is the perceived low competence in managing situations that are complex or difficult (e.g., handling pain and patients in a state of shock), which requires more attention on the part of the specific work place and the education system.
